# MicroRNA-4423-3p inhibits proliferation of fibroblast-like synoviocytes by targeting matrix metalloproteinase 13 in rheumatoid arthritis

**DOI:** 10.1080/21655979.2021.1988372

**Published:** 2021-11-29

**Authors:** Weihong Xu, Lu Ye, Huaxiang Wu

**Affiliations:** Department of Rheumatology, School of Medicine, The Second Affiliated Hospital of Zhejiang University, Hangzhou, China

**Keywords:** Rheumatoid arthritis, microRNA, mmp13, fibroblast-like synoviocytes

## Abstract

Rheumatoid arthritis (RA) is a chronic inflammatory autoimmune disease that is increasing in incidence worldwide. RA is regulated by a variety of microRNAs (miRNAs/miR). Moreover, analysis of public data has revealed that miR-4423-3p is significantly downregulated in peripheral blood mononuclear cells of RA patients. This study investigated the role of miR-4423-3p in RA. The levels of miR-4423-3p and matrix metalloproteinase 13 (MMP13) in RA patients and the regulatory relationship between miR-4423-3p and MMP13 were analyzed using public data. A dual-luciferase reporter assay was performed to verify that miR-4423-3p targets MMP13 in human fibroblast-like synoviocyte (HFLS) RA cells (HFLS-RA). Following the overexpression of miR-4423-3p, miR-4423-3p inhibitor, and MMP13 in HFLS-RA, viability, proliferation, cell cycle, apoptosis, and invasion/migration assays were used to detect the effects of miR-4423-3p targeting MMP13 on cell biological processes. The results revealed that miR-4423-3p was downregulated in peripheral blood mononuclear cells of RA patients and MMP13 was upregulated in synovial tissue of RA patients. miR-4423-3p targets the 3ʹ untranslated region of MMP13 and downregulates MMP13 expression. After overexpression of miR-4423-3p, cell proliferation, migration, and invasion were inhibited, the cell cycle was prevented and cell apoptosis was promoted. Overexpression of MMP13 promoted cell proliferation, migration, and invasion, while accelerating the cell cycle process and suppressing apoptosis. The findings indicate that in HFLS-RA cells, overexpression of miR-4423-3p inhibited proliferation, migration, and invasion, and promoted apoptosis by negatively regulating MMP13. The overexpression of miR-4423-3p might be a novel strategy for the treatment of RA.

## 1 Introduction

Rheumatoid arthritis (RA) is a chronic inflammatory disease characterized by symmetrical polyarthritis [1]. The pathological characteristics of RA are inflammation and hyperplasia of the synovial membrane of the joints, which are accompanied by erosion of bone and cartilage [1,2]. With the increase in life expectancy worldwide, the number of elderly people with RA is also increasing, and the incidence of the disease is increasing among younger people [3]. The disease seriously affects the work and life of patients, and reduces the quality of life and economic income of patients [3], which emphasizes the importance of early diagnosis and treatment.

In RA, human fibroblast-like synovial cells (HFLS) are the main component of synovitis. These cells (HFLS-RA) are the main players in arthritis due to their invasive and anti-apoptotic properties, as well as their ability to regulate immune responses and chronic inflammation [4]. In the joints of RA patients, HFLS-RA are greatly increased, resulting in thickened cell membranes. The cells may lead to degeneration and degradation of the outer matrix, which in turn erodes articular cartilage and bone [5]. Thus, HFLS-RA are the key cells in the pathogenesis of RA. Although the treatment of RA has improved, currently available disease-relief drugs do not directly target HFLS-RA dysfunction [6]. Therefore, novel and more effective treatment strategies need to be developed. Understanding the epigenetic properties of HFLS-RA may help in identifying new RA diagnostic and prognostic markers, targeting synovial fibroblasts, and regulating their cell biological behavior, which may be an effective way to treat RA [7]. At present, many studies have shown that non-coding RNA is abnormally expressed in HFLS-RA and plays a key role in the occurrence and development of RA, including microRNAs (miRNAs/miR) [8,9]. Many validated non-coding RNAs have been identified as promising biomarkers for the diagnosis and treatment of RA, such as miR-141, miR-4701-5p, miR-138, miR-410-3p [10–13].

miRNAs are a type of single-stranded non-coding RNA with a length of 18 to 25 nucleotides, which are mainly responsible for regulating gene expression [14]. These small molecules can regulate post-transcriptional gene expression levels by base pairing of two to eight nucleotides at the 5ʹ end (the ‘seed region’) [15]. Abnormal miRNA expression is associated with the pathogenesis of metabolic, cardiovascular, and autoimmune diseases and tumors. MiR-146a, miR-150, miR-155, and miR-233 may be involved in the development of RA [16]. MiRNAs can amplify the molecular mechanisms of inflammatory cytokines that include tumor necrosis factor-alpha (TNF-α); interleukin (IL)-1β, IL-6, and IL-17; proinflammatory mediators; growth factors; and the production of matrix metalloproteinases. Thus, miRNAs may be meaningful therapeutic targets [17].

Matrix metalloproteinase (MMP) 13 is mainly involved in cartilage degradation [18]. MMP13 is primarily expressed in connective tissues [19]. MMP13 levels are upregulated in patients with damaged articular cartilages, suggesting that increased MMP13 levels may be associated with cartilage degeneration [20]. Moreover, in cartilage, MMP13 degrades type II, IV, and IX collagen, proteoglycan, and osteonectin [21]. Transgenic mice overexpressing MMP13 develop a spontaneous articular cartilage destruction phenotype [22]. In addition, MMP13 promotes the migration of keratinocytes during wound healing and may also participate in the migration and invasion of tumor cells [23,24].

Although miR-19a reportedly participates in the degradation of chondrocytes during RA development by targeting MMP13 [25], the mechanism of the synergistic involvement of miRNAs and MMP13 in RA development remains unclear. In this study, to investigate other miRNAs targeting MMP13, we hypothesized that miR-4423-3p targets MMP13, and explored the effect of miR-4423-3p targeting MMP13 on cells using HFLS-RA to evaluate whether miR-4423-3p has the potential to be a therapeutic target for RA.

## 2 Methods

### 2.1 Bioinformatics analysis

Data of RA patients and healthy volunteers were downloaded from the Gene Expression Omnibus (GEO) database (GEO accession: GSE124373 and GSE77298) [26]. The data were analyzed using R software (version 3.5.1). The predicted mRNAs targeted by miR-4423-3p from TargetScan and the upregulated mRNAs in the synovial tissue of RA patients in the GEO database were visualized in a Venn diagram using the R software.

### 2.2 Plasmid construction

The open reading frame of the MMP13 gene (Gene ID: 4322) was cloned into the pCDH-EF1α-Flag-T2A-puro (Anti-HeLa BioTech, Xiamen, China) to construct the plasmid encoding the MMP13 protein and named MMP13 OE. The negative control of MMP13 OE is pCDH-EF1α-Flag-T2A-puro, named Vector. Wild-type or mutant 3ʹ untranslated region (UTR) of MMP13 was cloned into the pmriGLO vector (Anti-HeLa BioTech) to construct the plasmids, which were designated as MMP13 3ʹUTR WT and MMP13 3ʹUTR MUT. The primers used are listed in Table 1.

**Table 1.** Primers for plasmid construction

Name	Sequence (5′-3′)
MMP13 3ʹUTR WT-F	GCTCGCTAGCCTCGAGCTAAAGACTTGTATAGCATG
MMP13 3ʹUTR WT-R	ATGCCTGCAGGTCGACCACTTCCTAATACACTTTGC
MMP13 3ʹUTR MUT-F	CCCACACACGGATTTGATACTTGGGGAGGG
MMP13 3ʹUTR MUT-R	GTATCAAATCCGTGTGTGGGAAGTGCTGGG
MMP13-F	TAGAGCTAGCGAATTCATGCATCCAGGGGTCCTGGC
MMP13-R	CAGCGGCCGCGGATCCACACCACAAAATGGAATTTG
F: forward primer; R: reverse primer	

### 2.3 Cell culture

HFLS-RA purchased from Xiamen Immocell Biotechnology Co., Ltd. (Fujian, China) were cultured in Dulbecco’s modified Eagle’s medium (DMEM) supplemented with 10% fetal bovine serum (FBS) for subsequent analyses.

### 2.4 Dual-luciferase reporter assay

Negative control of miR-4423-3p mimic (mimic NC) or miR-4423-3p mimic (200 pmol/well), and MMP13 3ʹUTR WT or MMP13 3ʹUTR MUT plasmid (1 μg/well) were co-transfected into HFLS-RA in 24-well plates. After 48 h, the cells were lysed and the luciferase activity was detected using the Dual-Luciferase Reporter Assay System (Catalog number E1910, Promega, Madison, WI, USA.) [27].

### 2.5 Transfection

HFLS-RA were seeded onto the 6-well plates at a density of 1 × 10^6^ per well and were divided into seven group, mimic NC, miR-4423-3p mimic, inhibitor NC, miR-4423-3p inhibitor, mimic NC + Vector, miR-4423-3p mimic + Vector, and miR-4423-3p mimic + MMP13 OE. After 12 h, in mimic NC, miR-4423-3p mimic, inhibitor NC, and miR-4423-3p inhibitor groups, cells were transfected with 800 pmol/well mimic NC, miR-4423-3p mimic, inhibitor NC or miR-4423-3p inhibitor using Lipofectamine 2000 (Catalog number 11,668,019, Invitrogen), respectively. In mimic NC + Vector group, cells were transfected with 800 pmol/well mimic NC and 4 μg/well Vector. In miR-4423-3p mimic + Vector group, cells were transfected with 800 pmol/well miR-4423-3p mimic NC and 4 μg/well Vector. In miR-4423-3p mimic + MMP13 OE, cells were transfected with 800 pmol/well miR-4423-3p mimic NC and 4 μg/well MMP13 OE plasmid. After 48-h transfection, cells were collected for subsequent experiments.

### 2.6 Real-time PCR (RT-PCR)

After transfected as described above, HFLS-RA was treated using an RNA extraction kit (Catalog number 9767, TaKaRa Bio, Shiga, Japan) to extract total RNA which was reverse transcribed to cDNA using PrimeScript™ RT Master Mix (Catalog number RR036A, TaKaRa Bio). The obtained cDNA and TB Green® Fast qPCR Mix (Catalog number RR430A, TaKaRa Bio) were used to perform RT-PCR. U6 and 18s rRNA were used as internal controls. Relative expression levels were calculated using the comparative threshold cycle (2^−ΔΔCt^) method [28]. The primers used for RT-PCR are listed in Table 2.

**Table 2.** The primers for RT-PCR

Gene name	Primer name	Sequence (5′-3′)
U6	RT	AACGCTTCACGAATTTGCGT
	QF	CTCGCTTCGGCAGCACA
	QR	AACGCTTCACGAATTTGCGT
18s	QF	AGGCGCGCAAATTACCCAATCC
	QR	GCCCTCCAATTGTTCCTCGTTAAG
miR-4423-3p	RT	GTCGTATCCAGTGCAGGGTCCGAGGTATTCGCACTGGATACGACTTGTTG
	QF	CGCGATAGGCACCAAAAAG
	QR	AGTGCAGGGTCCGAGGTATT
MMP13	QF	CCTTGATGCCATTACCAGTCTCC
	QR	AAACAGCTCCGCATCAACCTGC
E-cadherin	QF	GCCTCCTGAAAAGAGAGTGGAAG
	QR	TGGCAGTGTCTCTCCAAATCCG
N-cadherin	QF	CCTCCAGAGTTTACTGCCATGAC
	QR	GTAGGATCTCCGCCACTGATTC
Vimentin	QF	AGGCAAAGCAGGAGTCCACTGA
	QR	ATCTGGCGTTCCAGGGACTCAT
Bax	QF	TCAGGATGCGTCCACCAAGAAG
	QR	TGTGTCCACGGCGGCAATCATC
Bcl-2	QF	ATCGCCCTGTGGATGACTGAGT
	QR	GCCAGGAGAAATCAAACAGAGGC
Cyclin D1	QF	TCTACACCGACAACTCCATCCG
	QR	TCTGGCATTTTGGAGAGGAAGTG
CDK2	QF	ATGGATGCCTCTGCTCTCACTG
	QR	CCCGATGAGAATGGCAGAAAGC

RT: reverse transcription; QF: forward primer for RT-PCR; QR: reverse primer for RT-PCR.

### 2.7 Cell viability assay

After transfected as described above, HFLS-RA were seeded in a 96-well plate (1.0 × 10^4^ cells/well). After 24, 48, and 72 h, 20 μL of 3-(4,5-dimethylthiazol-2-yl)-2,5-diphenyltetrazolium bromide (MTT; 5 mg/ml in PBS, pH 7.4) was added to each well for detecting cell proliferation as previously described [29]. After 4 h, 150 μL of DMSO was added to each well, and after shaking for 10 min, the absorbance values of each group were measured at 490 nm. The cell growth curve was plotted with the time as the abscissa and the ordinate of absorbance.

### 2.8 Cell proliferation assay using 5-ethynyl-2ʹ-deoxyuridine (EdU)

HFLS-RA, transfected as described above, were evenly seeded in wells of 96-well plates. After 24 h, the cells were treated with the Cell-Light EdU Apollo488 In Vitro Kit (Catalog number C10310-3, RiboBio, Guangzhou, China) according to the manufacturer’s instructions [29]. Nuclear DNA was stained with 5 μg/ml 4′,6-diamidino-2-phenylindole (DAPI; Catalog number C1002, Beyotime, Shanghai, China). Images were obtained using a fluorescent microscope (MOTIC, Hong Kong, China).

### 2.9 Cell cycle assay

After transfected as described above, HFLS-RA were harvested to examine the cell cycle as previously described [29]. In brief, the harvested cells were fixed in 70% ethanol at 4°C overnight. After incubated in PBS containing 0.2% Triton X-100 and 10 μg/mL RNase at 37°C for 30 min, the fixed cells were incubated with 20 μg/mL propidium iodide (PI) at room temperature for 30 min in the dark and then analyzed using a flow cytometer (ACEA Biosciences Inc., Hangzhou, China).

### 2.10 Cell apoptosis assay

After HFLS-RA were transfected as described above, adherent and floating cells were collected using 0.25% trypsin without Ethylene Diamine Tetraacetic Acid to test cell apoptosis using an Annexin V-FITC/PI Apoptosis Detection Kit (Catalog number A211-02, Vazyme, Nanjing, China) as previously described [29]. Subsequent analysis was performed using a flow cytometer (ACEA Biosciences Inc., Hangzhou, China).

### 2.11 Invasion/migration assay

Transwell plates (Catalog number 3464, Corning, New York, NY, USA) with or without Matrigel were used to measure invasion and migration, respectively, as previously described [29]. After were transfected as described above, 1 × 10^5^ HFLS-RA suspended in serum-free DMEM was seeded in the upper chambers of the Transwell plate, and 500 μL of DMEM containing 10% FBS was added on the lower chamber. After 24 h, migrated or invasive cells were stained with 0.5% crystal violet, and then the cells in six random fields were photographed and counted.

### 2.12 Western blotting

After transfected as described above, HFLS-RA were lysed in ice-cold RIPA buffer (Catalog number P0013 C, Beyotime) to extract protein for western blotting as previously described [30]. The protein was quantified using a BCA protein concentration assay kit (Catalog number P0012S, Beyotime). Subsequently, proteins were separated by 10% denaturing SDS-PAGE. After separation, the proteins were transferred to PVDF membranes. After incubation with 5% skim milk at 28°C for 2 h, membranes were incubated with the primary antibody for 2 h at room temperature, followed by incubation with the appropriate secondary antibody for 1 h at room temperature. After the excess antibodies on the membrane were washed off, electrochemiluminescence (ECL) was used to detect immune complexes. The protein gray values were analyzed using ImageJ software (NIH, Bethesda, MD, USA). The antibodies used for western blotting are listed in Table 3.

**Table 3.** Antibodies for western blotting

Classification	Name of antibody	Manufacturer	Catalog No.	Dilution rate
Primary antibody	Bcl-2 antibody	Cell signaling technology	15,071	1:1000
	Bax antibody	Cell signaling technology	89,477	1:1000
	Cyclin D1 antibody	Proteintech	26,939-1-AP	1:1000
	CDK2 antibody	Proteintech	10,122-1-AP	1:1000
	GAPDH antibody	Proteintech	10,494-1-AP	1:5000
	MMP13 antibody	Proteintech	18,165-1-AP	1:1000
	E-Cadherin antibody	Proteintech	60,335-1-Ig	1:2000
	N-Cadherin antibody	Proteintech	66,219-1-Ig	1:2000
	Vimentin antibody	Proteintech	60,330-1-Ig	1:10,000
Secondary antibody	HRP-conjugated Goat Anti-Rabbit IgG	Proteintech	SA00001-2	1:5000
	HRP-conjugated Goat Anti-mouse IgG	Proteintech	SA00001-1	1:5000

### 2.13 Statistical analyses

All statistical analyses were performed using SPSS software (version 22.0; IBM SPSS, Armonk, NY, USA). The comparison of non-parametric data between the two groups was performed using the Mann-Whitney test. One-way analysis of variance (ANOVA) was used to identify significant differences among multiple groups, followed by Tukey’s post hoc test. The parametric data between the two groups were compared using the student’s t-test (unpaired). Statistical significance was set at *P* < 0.05.

## 3 Results

Here, we aimed to investigate the role of miR-4423-3p targeting MMP13 in HFLS-RA. We performed a series of bioinformatics analysis and *in vitro* assays, and found that miR-4423-3p inhibited proliferation, migration, and invasion, and promoted apoptosis by negatively regulating MMP13 in HFLS-RA. Therefore, our data for the first revealed the functional roles of miR-4423-3p in HFLS-RA, providing new insights into the pathogenesis and potential therapeutic targets of RA.

### 3.1 miR-4423-3p inhibits proliferation of HFLS-RA

**Figure 1.**
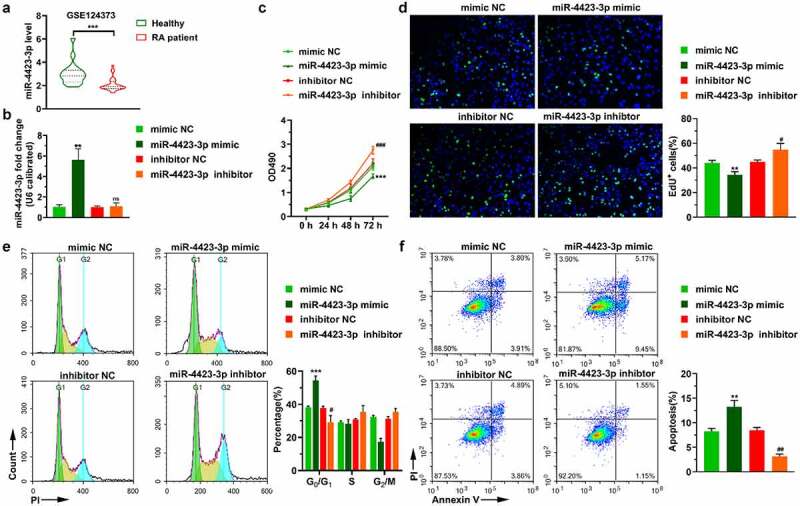
**miR-4423-3p inhibits proliferation of HFLS-RA. A**: The levels o**f** miR-4423-3p were differentially expressed in healthy people (n = 18) and RA patients (n = 28) in data from the GEO database (GSE124373); ***P < 0.001. **B**: Intracellular miR-4423-3p expression changes in different groups. **C**: The MTT assay was used to determine the changes of cell viability in each group. **D**: Representative images of EdU assay (left) and statistical histograms of EdU^+^ cells (right). **E**: Representative images of cell cycle assay (left) and statistical histograms of cell proportions in different phases (right). **F**: Representative image of apoptosis assay and statistical histogram of the proportion of apoptotic cells in different groups. Mann-Whitney test (a) and student’s t test (b–f) were used for statistical analysis. All experiments were carried out independently at least three times. PI: propidium iodide. **P < 0.01, ***P < 0.001 vs. mimic NC; ns: not significant, #*P* < 0.05, ##*P* < 0.01, ###*P* < 0.001 vs. inhibitor NC

The GEO database (GSE124373) was used to analyze expression changes of miR-4423-3p in peripheral blood mononuclear cells of healthy individuals and patients with RA. MiR-4423-3p was significantly downregulated in peripheral blood mononuclear cells of RA patients (*P* < 0.0001) (Figure 1(a)). HFLS-RA were transfected with miR-4423-3p mimic, mimic NC, inhibitor NC, or miR-4423-3p inhibitor. The miR-4423-3p expression levels of cells in each group were analyzed by RT-PCR. Compared with the mimic NC group, miR-4423-3p expression was significantly increased in the miR-4423-3p mimic group (*P* < 0.01). Furthermore, there was no significant difference in the level of miR-4423-3p between the inhibitor NC and miR-4423-3p inhibitor groups, suggesting that the miR-4423-3p inhibitor did not degrade miR-4423-3p (Figure 1(b)). The results of the MTT and EdU assays indicated that the miR-4423-3p mimic inhibited cell proliferation, while the miR-4423-3p inhibitor promoted cell proliferation (Figures 1(c,d)). Cell cycle assay results showed that the overexpression of miR-4423-3p arrested the cell cycle in the G_0_/G_1_ phase, while inhibition of miR-4423-3p accelerated cell cycle progression (Figure 1(e)). The apoptosis assay showed that upregulation of miR-4423-3p increased the proportion of apoptotic cells, while inhibition of miR-4423-3p decreased the proportion of apoptotic cells (figure 1(f)).

These collective results indicated that miR-4423-3p was significantly downregulated in the RA patient population. In vitro, the overexpression of miR-4423-3p lead to significant inhibition of cell proliferation, arrest of the cell cycle in the G_0_/G_1_ phase, and the promotion of apoptosis.

### 3.2 miR-4423-3p suppresses HFLS-RA migration and invasion

**Figure 2.**
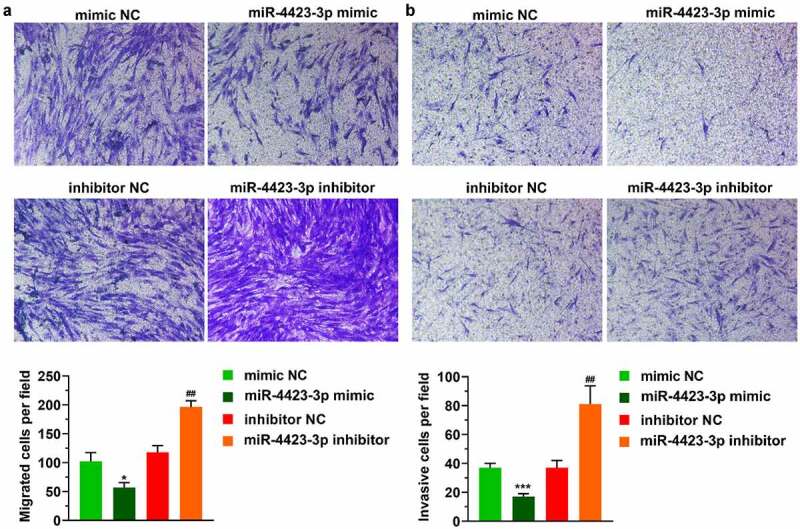
**miR-4423-3p suppresses migration and invasion of HFLS-RA. A**: Representative images of migrated cells (up) and statistical histograms of the number of migrated cells in per field in different groups (bottom). **B**: Representative images of invasive cells (up) and statistical histograms of the number of invasive cells in per field in different groups (bottom). Student’s t test (a and b) was used for statistical analysis. All experiments were carried out independently at least three times. *P < 0.05, ***P < 0.001 vs. mimic NC; ##*P* < 0.01 vs. inhibitor NC

In the Transwell assay, the numbers of migrating and invading cells were significantly reduced in the miR-4423-3p mimic group. After the inhibition of miR-4423-3p, the numbers of migrated and invasive cells were significantly enhanced (Figures 2(a,b)). These results suggested that upregulation of miR-4423-3p inhibited cell migration and invasion.

### 3.3 MMP13 is a direct target of miR-4423-3p

**Figure 3.**
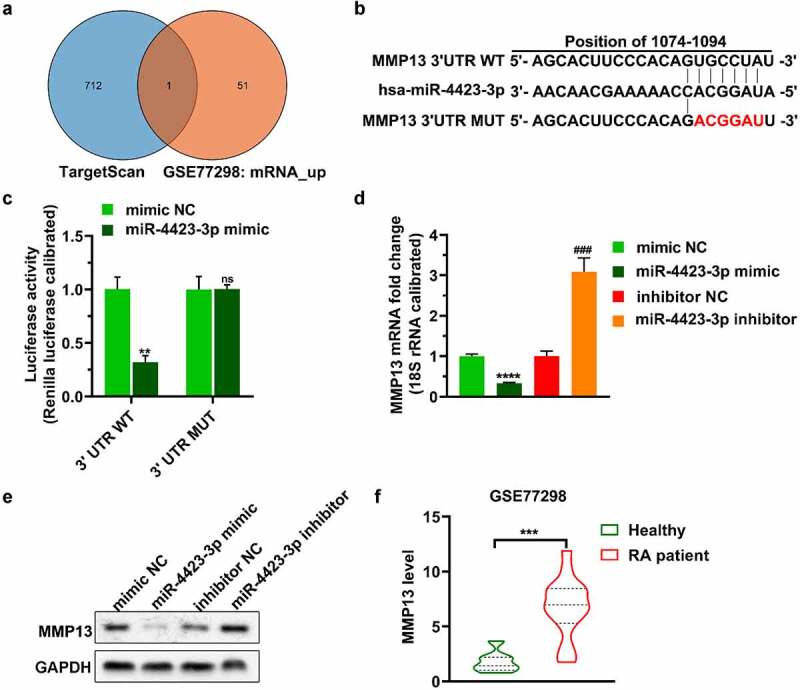
**MMP13 is a direct target of miR-4423-3p. A**: Venn diagram of mRNAs targeted by miR-4423-3p. **B**: Schematic diagram of the 3ʹUTR mutation site of MMP13. **C**: Dual-luciferase reporter assay verified the targeting relationship between miR-4423-3p and MMP13. Ns: not significant, **P < 0.01 vs. mimic NC. **D and E**: After overexpression or inhibition of miR-4423-3p in HFLS-RA, mRNA and protein levels of MMP13 were detected by RT-PCR (d) and western blotting (e), respectively. ****P < 0.0001 vs. mimic NC; ###*P* < 0.001 vs. inhibitor NC. **F**: Analysis of the difference of MMP13 levels between RA patients (n = 16) and healthy volunteers (n = 7) from GSE77298; ***P < 0.001. Student’s t test (c and d) and Mann-Whitney test (f) were used for statistical analysis. All experiments were carried out independently at least three times

The predicted mRNAs targeted by miR-4423-3p in TargetScan and the mRNAs upregulated in GSE77298 were plotted as a Venn diagram. One mRNA (MMP13) was included in both databases (Figure 3(a)). Subsequently, a dual-luciferase reporter assay verified that miR-4423-3p targeted the 3ʹUTR of MMP13 (Figures 3(b,c)). Upregulation of miR-4423-3p decreased the levels of MMP13 mRNA and protein, while inhibition of miR-4423-3p increased the levels of MMP13 mRNA and protein (Figures 3(d,e)). Analysis of the GSE77298 data revealed higher levels of MMP13 in synovial tissue of RA patients than the levels in synovial tissue of healthy volunteers (figure 3(f)).

### 3.4 Replenishing MMP13 promotes cell proliferation and decreases apoptosis

**Figure 4.**
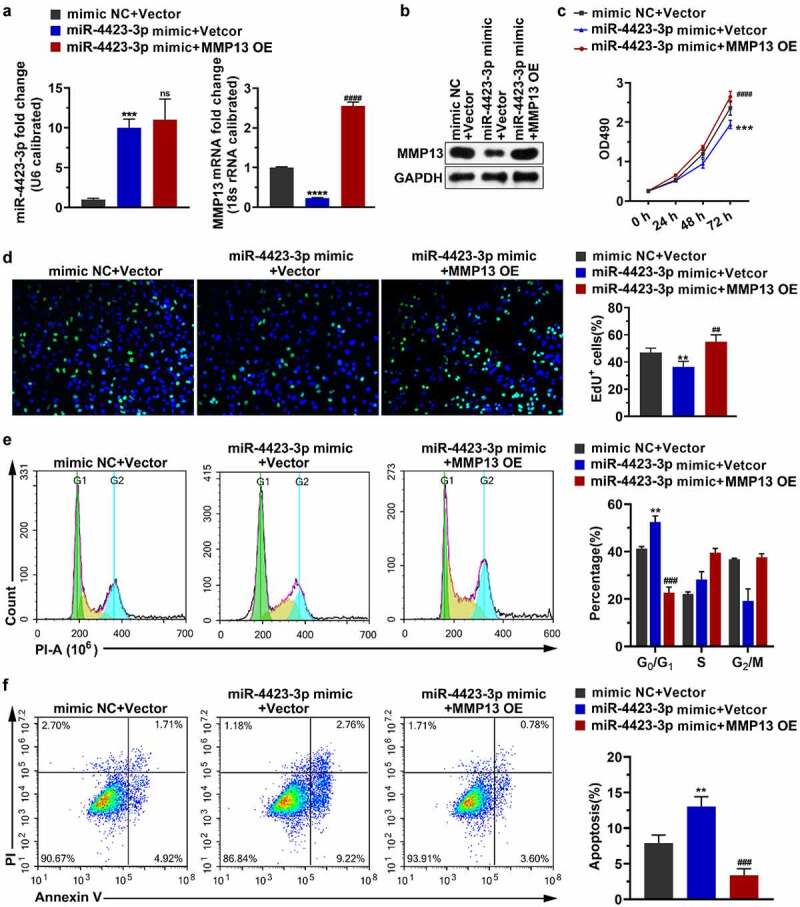
**Replenishing MMP13 promotes cell proliferation and decreases apoptosis**. The miR-4423-3p mimic and MMP13 OE plasmid were co-transfected into HFLS-RA. **A**: The RNA levels of miR-4423-3p and MMP13 were tested by RT-PCR. **B**: The protein level of MMP13 was detected by western blotting. **C and D**: Cell proliferation was analyzed using MTT (c) and EdU (d) assays. **E**: Cell cycle was detected using PI staining. **F**: Apoptosis was analyzed using Annexin V-FITC/PI staining. One-way ANOVA (A‒F) was used for statistical analysis. All experiments were carried out independently at least three times. PI: propidium iodide; mimic NC + Vector: mimic NC and pCDH-EF1α-Flag-T2A-puro vector were co-transfected into HFLS-RA; miR-4423-3p mimic + Vector: miR-4423-3p mimic and pCDH-EF1α-Flag-T2A-puro vector co-transfected into HFLS-RA; miR-4423-3p mimic + MMP13 OE: miR-4423-3p mimic and MMP13 OE plasmid co-transfected into HFLS-RA. **P < 0.01, ***P < 0.001, ****P < 0.0001 vs. mimic NC + Vector; ns: not significant, ##*P* < 0.01, ###*P* < 0.001, ####*P* < 0.0001 vs. miR-4423-3p mimic + Vector

Transfection of the miR-4423-3p mimic into HFLS-RA upregulated the level of miR-4423-3p and downregulated the mRNA and protein levels of MMP13. Simultaneous transfection of the miR-4423-3p mimic and the MMP13 OE plasmid into HFLS-RA restored the mRNA and protein levels of MMP13 (Figures 4(a,b)). Replenishing MMP13 alleviated the inhibitory effect of miR-4423-3p on cell proliferation (Figures 4(c,d)), the cell cycle arrest by miR-4423-3p (Figure 4(e)), and the induction of apoptosis by miR-4423-3p (figure 4(f)). These findings revealed that miR-4423-3p inhibited cell proliferation and induced apoptosis by negatively regulating MMP13 expression.

### 3.5 miR-4423-3p suppresses HFLS-RA migration and invasion by targeting MMP13

**Figure 5.**
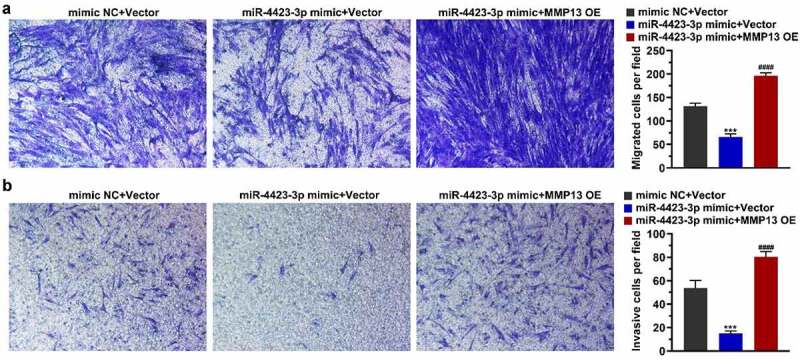
**miR-4423-3p suppresses migration and invasion of HFLS-RA by targeting MMP13. A**: Representative images of migrated cells (left) and statistical histograms of the number of migrated cells in per field in different groups (right). **B**: Representative images of invasive cells (left) and statistical histograms of the number of invasive cells in per field in different groups (right). One-way ANOVA (a and b) was used for statistical analysis. All experiments were carried out independently at least three times. mimic NC + Vector: mimic NC and pCDH-EF1α-Flag-T2A-puro vector co-transfected into HFLS-RA; miR-4423-3p mimic + Vector: miR-4423-3p mimic and pCDH-EF1α-Flag-T2A-puro vector co-transfected into HFLS-RA; miR-4423-3p mimic + MMP13 OE: miR-4423-3p mimic and MMP13 OE plasmid co-transfected into HFLS-RA. ***P < 0.001 vs. mimic NC + Vector; ####*P* < 0.0001 vs. miR-4423-3p mimic + Vector

After the miR-4423-3p mimic and MMP13 OE plasmid were co-transfected into HFLS-RA, a Transwell assay was used to detect cell migration and invasion. Overexpression of miR-4423-3p inhibited cell migration and invasion, while the simultaneous overexpression of miR-4423-3p and MMP13 abrogated the inhibitory effect of miR-4423-3p on migration and invasion. (Figure 5). These results suggested that overexpression of MMP13 rescued the inhibition of cell migration and invasion caused by miR-4423-3p.

### 3.6 miR-4423-3p regulates expression of genes related to apoptosis, cell cycle and epithelial-mesenchymal transition (EMT) by targeting MMP13

**Figure 6.**
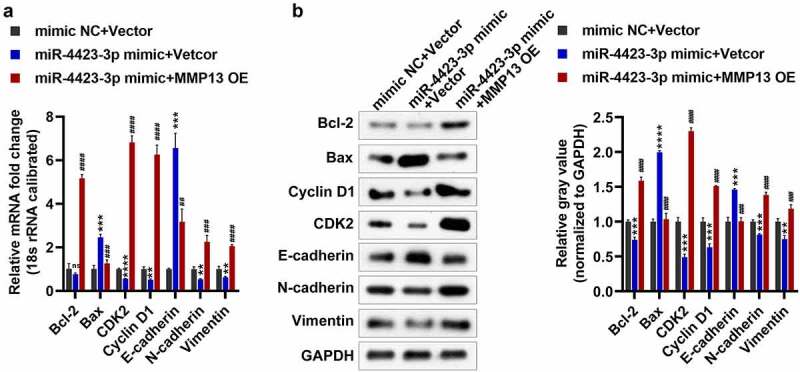
**Detection of apoptosis, cell cycle and EMT-related genes. A**: The mRNA levels of Bax, Bcl-2, CDK2, Cyclin D1, E-cadherin, N-cadherin, and Vimentin were tested by RT-PCR. **B**: The protein levels of Bax, Bcl-2, CDK2, Cyclin D1, E-cadherin, N-cadherin, and vimentin were tested by western blotting. One-way ANOVA (a and b) was used for statistical analysis. All experiments were carried out independently at least three times. mimic NC + Vector: mimic NC and pCDH-EF1α-Flag-T2A-puro vector co-transfected into HFLS-RA; miR-4423-3p mimic + Vector: miR-4423-3p mimic and pCDH-EF1α-Flag-T2A-puro vector co-transfected into HFLS-RA; miR-4423-3p mimic + MMP13 OE: miR-4423-3p mimic and MMP13 OE plasmid co-transfected into HFLS-RA. ns: not significant, **P < 0.01, ***P < 0.001, ***P < 0.001 vs. mimic NC + Vector; ##*P* < 0.01, ###*P* < 0.001, ####*P* < 0.0001 vs. miR-4423-3p mimic + Vector

As a preliminarily investigation of the molecular mechanism of the regulation of cell apoptosis, cell cycle, migration, and invasion by the targeting of MMP13 by miR-4423-3P, we tested the expression of marker genes related to apoptosis (Bax and Bcl-2), cell cycle (Cyclin D1 and CDK2), and EMT (E-cadherin, N-cadherin, and Vimentin). Overexpression of miR-4423-3p increased the mRNA and protein levels of Bax and E-cadherin, and significantly reduced the protein levels of Bcl-2, and the mRNA and protein levels of CDK2, cyclin D1, N-cadherin, and vimentin. However, supplementation with MMP13 significantly decreased the mRNA and protein levels of Bax and E-cadherin, while increasing the mRNA and protein levels of Bcl-2, CDK2, cyclin D1, N-cadherin, and vimentin (Figure 6). These findings suggested that miR-4423-3p regulates apoptosis, cell cycle, and EMT-related marker gene expression by targeting MMP13, thereby regulating apoptosis, cell cycle, migration, and invasion.

## 4 Discussion

In recent years, research has begun to focus on the mechanisms of epigenetics in autoimmune diseases. miRNAs bind to messenger RNAs to affect target gene expression and affect disease progression [31]. The research on epigenetic correlation of RA has mainly focused on miRNAs [32]. Genetic variation in some miRNA genes induces the malignant progression of RA, and miRNA expression also varies depending on the stage and activity of the disease. Some miRNA analogs or antagonists have the potential to treat RA [33]. These findings indicate that revealing the changes in the expression level of miRNAs in RA is helpful to understand the pathogenesis of RA and to provide new strategies for the diagnosis and treatment of RA. In this study, the level of miR-4423-3p in peripheral blood monocytes of RA patients was significantly reduced compared to that in healthy controls. Furthermore, we verified the role of miR-4423-3p in the proliferation, migration, and invasion of HFLS-RA and identified its mRNA target. The findings will facilitate studies aiming to reveal the pathogenesis of RA and improve its diagnosis and treatment.

A report pointed out that the bacteria on the mucosal surface of human intestines, gums and respiratory system are sufficient to change the local and systemic host immune response and cause joint inflammation, and there is evidence that the lung mucosa may be the early site of autoimmune-related injury and/or may be the site of RA-related autoimmunity [34]. Furthermore, as a miRNA that is unique to primates, miR-4423 is mainly expressed in airway epithelial cells and regulates airway epithelial differentiation and inhibits the occurrence of lung cancer [35]. These results indicate that miR-4423 is likely to play a role in the occurrence and development of RA, which was just confirmed in this study. Doxorubicin reportedly induces the upregulation of miR-4423-3p in human-induced pluripotent stem cell-derived cardiomyocytes [36]. Moreover, the genes targeted by miR-4423-3p are mostly enriched in myocardial specific pathways, such as myocardial contraction [36]. In patients with rheumatic heart disease, miR-4423-3p expression is significantly downregulated [37]. However, the role of miR-4423 in RA has rarely been reported. In the present study, miR-4423-3p expression was significantly downregulated in RA patients. An increased miR-4423-3p expression inhibited proliferation, migration, and invasion, while arresting the cell cycle in the G_0_/G_1_ phase and promoting apoptosis. These results suggest that elevated miR-4423-3p expression levels may inhibit the development of RA.

In RA, MMPs that are induced by inflammatory cytokines, including IL-1 beta and TNF-α, degrade all components of the extracellular matrix [38]. Since the MMP-1 and MMP-13 collagenases are the rate-limiting enzymes in the process of collagen degradation, they play a major role in RA [38]. Synovial cells that connect joints produce MMP-1, whereas chondrocytes in cartilage produce MMP-13 [38–40]. We presently observed that the transcription level of MMP13 in the synovial tissue of RA patients was significantly upregulated, indicating that synovial cells may also produce MMP-13.

Studies have shown that the expression of MMP13 in synovial cells is regulated by multiple miRNAs, thereby affecting cell proliferation and invasion [25,41]. For example, miR-19a inhibits the proliferation and invasion of HFLS-RA by downregulating the expression level of MMP13 [25]. Similarly, we found in this study that MMP13 was a target gene of miR-4423-3p, which was confirmed by the dual-luciferase assay. Furthermore, miR-4423-3p negatively regulated MMP13 in HFLS-RA. Upregulation of MMP13 alleviated the inhibitory effect of miR-4423-3p on the proliferation, migration, and invasion of HFLS-RA and the apoptosis induced by miR-4423-3p. Increasing miR-4423-3p upregulated the expression of Bax and E-cadherin, and downregulated the expression of Bcl-2, CDK2, cyclin D1, N-cadherin, and vimentin. Supplementation with MMP13 reversed the regulation of miR-4423-3p on the expression of these genes. The collective findings indicate that miR-4423-3p regulates apoptosis, cell cycle, and EMT-related marker gene expression by targeting MMP13, thereby regulating apoptosis, cell cycle, migration, and invasion of HFLS-RA. Therefore, miR-4423-3p has the potential to treat RA by targeting MMP13.

This study only clarified that miR-4423-3p regulates the proliferation of HFLS-RA by targeting MMP13. Due to the lack of research on the role of miR-4423-3p in inflammation and animal experiments, the precise role of miR-4423- 3p in the treatment of RA was not revealed, which is a limitation of this study.

## 5 Conclusions

In summary, this study verified that overexpression of miR-4423-3p regulates the expression of apoptosis, cell cycle, and EMT-related marker genes by downregulating the expression of MMP13, thereby inhibiting the proliferation, migration, and invasion of HFLS-RA. Upregulation of miR-4423-3p has the potential to become a novel strategy for the treatment of RA.

## Data Availability

The data that support the findings of this study are available from the corresponding author upon reasonable request.
